# A promising perovskite primary explosive

**DOI:** 10.1038/s41467-023-43320-0

**Published:** 2023-11-27

**Authors:** Yongan Feng, Jichuan Zhang, Weiguo Cao, Jiaheng Zhang, Jean’ne M. Shreeve

**Affiliations:** 1 School of Environment and Safety Engineering, North University of China, 030051 Taiyuan, China; 2 Department of Chemistry, University of Idaho, Moscow, ID 83844-2343 USA; 3 Sauvage Laboratory for Smart Materials, Harbin Institute of Technology, 518055 Shenzhen, China

**Keywords:** Energy, Metal-organic frameworks

## Abstract

A primary explosive is an ideal chemical substance for performing ignition in military and commercial applications. For over 150 years, nearly all of the developed primary explosives have suffered from various issues, such as troublesome syntheses, high toxicity, poor stability or/and weak ignition performance. Now we report an interesting example of a primary explosive with double perovskite framework, {(C_6_H_14_N_2_)_2_[Na(NH_4_)(IO_4_)_6_]}_n_ (**DPPE-1**), which was synthesized using a simple green one-pot method in an aqueous solution at room temperature. **DPPE-1** is free of heavy metals, toxic organic components, and doesn’t involve any explosive precursors. It exhibits good stability towards air, moisture, sunlight, and heat and has acceptable mechanical sensitivities. It affords ignition performance on par with the most powerful primary explosives reported to date. **DPPE-1** promises to meet the challenges existing with current primary explosives, and this work could trigger more extensive applications of perovskite.

## Abstract (web summary)

A primary explosive (DPPE-1) with a double perovskite structure and ignition function was reported. It is synthesized in a straightforward manner and has good stability, with a minimum primary charge of 5 mg.

## Introduction

Primary explosives are a class of high-energy materials that perform precise ignition or start-up in commercial, military, and space exploration applications. Over the past 150 years, countless numbers of energetic substances have been designed and screened as possible initiating explosives, including transition metal-based, potassium-based, and organics^1^. However, transition metal-based substances suffer from toxic heavy metal and virulent organic precursors^2–9^, potassium-based substances have the problems of tedious synthesis (high cost) and weak ignition^10–18^, while organic substances are unstable and have troublesome preparations (e.g., toxic solvents and dangerous reactions)^19–22^. It seems an impossible but attractive challenge to develop primary explosives with green, low cost, and powerful ignition performance including acceptable stability.

**Fig. 1:**
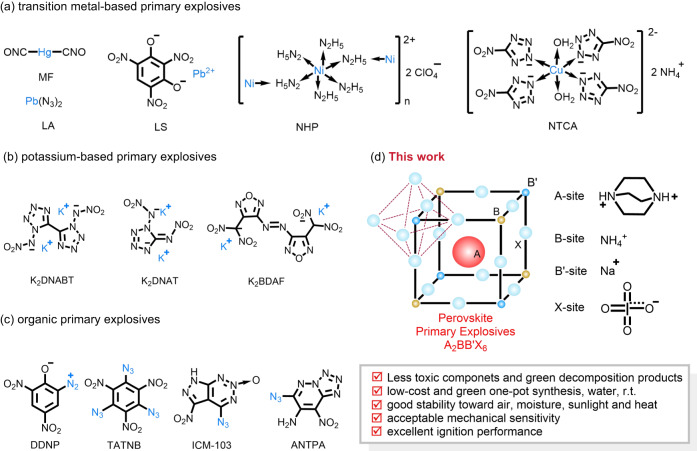
Several types of primary explosives: traditional and our reported ones. **a** MF is mercury fulminate^3,4^. LA is lead azide (PbN_6_)^3,4^. LS is lead styphnate (PbC_6_HN_3_O_8_)^3,4^. NHP is nickel hydrazine perchlorate ([Ni(N_2_H_4_)_3_](NO_3_)_2_)^5^. NTCA is 5-nitrotetrazolato copper ammonium ((NH_4_)_2_[Cu^II^(NT)_4_(H_2_O)_2_])^4^. **b** K_2_DNABT is dipotassium 1,1’-dinitramino-5,5’-bistetrazolate^10^. K_2_DNAT is dipotassium 1,5-di(nitramino)tetrazolate^12^. K_2_BDAF is dipotassium 4,4’-bis (dinitromethyl)-3,3’-azofurazanate^14^. **c** DDNP is 2-diazo-4,6-dinitrophenol^20^. TATNB is 1,3,5-triazido-2,4,6-trinitrobenzene^4^. ICM-103 is 6-nitro-7-azido-pyrazol[3,4-d][1,2,3]triazine-2-oxide^20^. ANTPA is 6-azido-8-nitro-tetrazolo[1,5-b]pyridazine -7-amine^21^. **d** This study reports an energetic perovskite with the general formula A_2_BB’X_6_, where A is 1,4-diazabicyclo[2.2.2]octane (or dabconium, or DABCO^2+^, or H_2_dabco^2+^), B is sodium (Na^+^), B’ is ammonium (NH_4_^+^), and X is periodate (IO_4_^−^).

In response to the above challenge, an A_2_BB’X_6_-type perovskite initiating substance {(C_6_H_14_N_2_)_2_[Na(NH_4_)(IO_4_)_6_]}_n_ (**DPPE-1**), in which the structure is very different from those of traditional primary explosives was developed (Fig. 1). Moreover, it shows a series of advantages, such as being free of highly toxic components, simple and green synthesis, good stability towards air, moisture, sunlight, heat and mechanical stimulation, and high ignition performance, demonstrating that perovskites with reasonable design have obvious advantages in the development of advanced primary explosives.

Recently, there has been intense interest in the broad family of materials based on perovskite frameworks^23–30^. Interest in these systems derives from the confluence of low-cost solution processing, chemical and functional diversity, and tunable structure and properties^31–33^. In addition, reasonably designed perovskites can also eliminate toxicity effectively, and improve stability under ambient conditions^34–37^. Primary explosives that topologically mimic perovskites are likely to be a new generation of primary explosives. A previous studies have reported several sensitive energetic perovskites (H_2_A)[Ag(ClO_4_)_3_] and indicated that they may be potential primary explosives^38,39^. However, the lack of data on ignition performance makes it highly uncertain whether these substances can be used as primary explosives. Moreover, these silver-based perovskites cannot avoid heavy metal pollution and unaffordable costs (noble metal Ag). Therefore, we started the study of perovskite primary explosives with advantages of green, low cost and high ignition performance. The focus is on the suitable chemical composition which can magically change the properties of a perovskite giving rise to a desired ignition function. For a perovskite framework, it is reasonable to configure a monovalent periodate anion (IO_4_^−^) at the X site, since it has both a powerful oxidizing ability to meet a desired high ignition performance as well as an acceptable sensitivity to realize reliable ignition. Our previous studies have confirmed that IO_*n*_^−^-based energetic substances exhibit strong oxidizing, high sensitivity, and rapidly exothermic behavior with iodine, which exhibits a bactericidal effect and is bio-friendly as the decomposition product^39–41^, thus showing great potential as green primary explosives. The latest research also shows the possibility of periodate-based primary explosives^42,43^. In addition, periodate (IO_4_^−^) tends to form three-dimensional frameworks through inter-ion interactions (Supplementary Fig. 1). However, currently reported initiating substances all belong to single-perovskite energetic materials, while double-perovskite energetic materials have not been reported yet, especially the existing synthesis methods of periodate-based perovskites are uneconomical and dangerous^43^, and their ignition performance still needs to be improved^43^. Now, we have reported a double-perovskite primary explosive (A_2_BB’X_6_ form) with excellent ignition performance. Non-toxic cations Na^+^ and NH_4_^+^ are selected to be located at sites B and B’, respectively, since both of them beneficially give rise to a compact structure, enhance biocompatibility and assist the reaction in proceeding in aqueous solution. As for the A site, the size of the framework formed by the interconnection of Na^+^ (or NH_4_^+^) and IO_4_^−^ is estimated to be ~7.42 Å × 7.42 Å × 7.42 Å^44,45^. To obtain the maximum crystal density and filling coefficient, dabconium (H_2_dabco^2+^, C_6_H_14_N_2_^2+^) with an effective radius 3.39 Å is preferred among a series of reported organic amine cations^46^. All involved cations and anions are common stable substances which are environmentally friendly. They are expected to be held together in aqueous solution to form the target organic–inorganic perovskite by simple self-assembly reaction based on interion interactions.

## Results

### Synthesis and crystalline structure

**Fig. 2:**
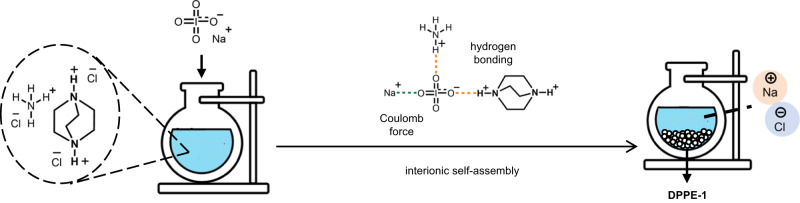
The low-cost and green synthesis of DPPE-1. Dabconium dihydrochloride (H_2_dabcoCl_2_, 1 M) and ammonium chloride (NH_4_Cl, 2 M) were dissolved in water by vigorous stirring at room temperature, then the aqueous solution of sodium metaperiodate (NaIO_4_, 6 M) was added to the above mixed solution, and the target substance DPPE-1 quickly precipitated within a few seconds (yield: >70%). The synthesis of the energetic double perovskite is a typical self-assembly process, which involves three inter-ionic interactions: hydrogen bonding between H_2_dabco^2+^ and IO_4_^−^, hydrogen bonding between NH_4_^+^ and IO_4_^−^, and coulomb force between H_2_dabco^2+^, NH_4_^+^ and IO_4_^−^.

With the above considerations in mind, the synthesis of the perovskite primary explosive was undertaken. The synthetic process of {(C_6_H_14_N_2_)_2_[Na(NH_4_)(IO_4_)_6_]}_n_ (**DPPE-1**) is very simple, green, and economical, namely, NaIO_4_, NH_4_Cl and dabconium dihydrochloride (H_2_dabcoCl_2_) were introduced into water and stirred at room temperature, resulting in the precipitation of a large amount of white solid within seconds. This gave rise to a granular crystalline product in good yield (72.1 wt%) after filtration to leave a clear colorless solution (Fig. 2 and Supplementary Fig. 2). Its structure has been characterized by elemental analysis, infrared spectrum, nuclear magnetic resonance, and single-crystal X-ray diffraction (Supplementary Figs. 3–8).

Single-crystal X-ray diffraction determination shows that DPPE-1 crystallizes in a structure in the *cubic* space group *Pa-3* having four formula units per unit cell (*a* **=** *b* **=** *c* **=** 14.8 Å) and a high crystal density of 2.89 g cm^−3^ at 120 K (Supplementary Table 1). With H_2_dabco^2+^ as an A-site cation, NH_4_^+^ as a B-site cation, Na^+^ as a B´-site cation, and IO_4_^−^ as the X-bridge, the structure of DPPE-1 can be accurately described as an A_2_BB’X_6_-type double perovskite, which is different from all those reported energetic materials with ABX_3_-type single-perovskite structure^25,47–50^. The experimental elemental analysis shows that the composition of DPPE-1 is 10.14% for C, 2.19% for H, and 4.89% for N, respectively (Supplementary Information 3. Synthesis), which is highly consistent with the theoretical calculation obtained from the double-perovskite substance, confirming the formation of DPPE-1 rather than reported (H_2_dabco)[Na(IO_4_)_3_] (DAI-1), (H_2_dabco)[(NH_4_)(IO_4_)_3_] (DAI-4) or (H_2_dabco)[Na(H_4_IO_6_)_3_] (DAI-X1). In terms of composition, DPPE-1 is similar to some energetic perovskites already reported, such as (H_2_dabco)[Na(ClO_4_)_3_] (DAP-1), (H_2_dabco)[(NH_4_)(ClO_4_)_3_] (DAP-4), DAI-1, DAI-4, and DAI-X1. However, it would be a great mistake to classify them as very close analogues. In most fields, the order of magnitude increase caused by structural changes means more interesting properties and functions. For single perovskites, there are theoretically *N*^3^ (*N* **=** 1,2,3,……*n*) molecular combinations, while for double perovskites, the variations could be extended to *N*^4^ (*N* **=** 1,2,3,……n). Obviously, double perovskite energetic materials own more variations, which provides good opportunities for the discovery of some new functions and applications. Interestingly, we also noted that DAI-X1 and DAI-4 were respectively formed in the absence of ammonium ions and sodium ions, while DPPE-1 was synthesized in the presence of both ions, which demonstrated the structural and functional diversity of energetic perovskites.

**Fig. 3:**
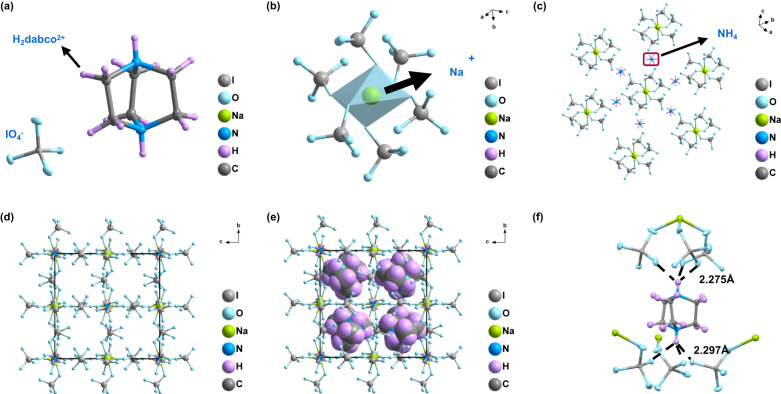
Hierarchical self-assembly of DPPE-1 with well-defined primary, secondary, tertiary, and quaternary structures. **a** Primary structures of the metaperiodate IO_4_^−^ anion and H_2_dabco^2+^. **b** Secondary structural unit (*Octa-small*) composed of Na^+^ and IO_4_^−^; based on the coordination bond (Na···O) and *Coulombic* forces (Na + /IO_4_^−^). **c** Tertiary structural unit (*Octa-large*) assembled by NH_4_^+^, for which a twisted octahedral configuration (*Octa-small*) occurs through interionic interactions (hydrogen bonding and *Coulombic* forces). **d** Unfilled cubic framework. **e** Cubic framework filled with H_2_dabco^2+^ cations. **f** Hydrogen bonds between the four twisted octahedral configurations (*Octa-small*) and the H_2_dabco^2+^ cations.

**DPPE-1** exhibits a hierarchical self-assembly configuration with well-defined primary, secondary, tertiary, and quaternary structures (Fig. 3). In the unit cell, each Na^+^ is bonded to six IO_4_^−^ ions via coordination bonds (Na···O) and Coulombic forces (Na^+^/IO_4_^−^), forming a twisted octahedral configuration (Fig. 3b, *Octa-small*), which is then connected with six adjacent NH_4_^+^ ions through hydrogen bonds (N-H···O, 2.257 Å) and Coulombic forces (NH_4_^+^/IO_4_^−^) to form a regular, larger octahedral structure (Fig. 3c, *Octa-large*). As the *Octa-large* continues to expand along the three axes based on the interionic interactions, a regular cubic framework with eight cavities is eventually formed (Fig. 3d), each of which is occupied by a dabconium cation (H_2_dabco^2+^) (Fig. 3e). With the assistance of the hydrogen bonds (N-H···O), each H_2_dabco^2+^ is completely connected to four neighboring *Octa-small* creating a unique double tetrahedral configuration (Fig. 3f), resulting in the calculated filling coefficient as high as 80.7% (Supplementary Fig. 9), which may be the highest in the field of high-energy materials to date.

Interestingly, all the observed hydrogen bonds are H···O interactions, and most of the bond lengths are less than expected. For example, the bond lengths of N-H···O and C-H···O are 2.21–2.46 Å and 2.41–2.60 Å (Supplementary Table 6), whereas the expected bond length is 2.46 Å, making these hydrogen bonds relatively short from a statistical point of view^51^. We think that the synergistic effect of coulomb forces may play a positive role. Notably, both NH_4_^+^ and H_2_dabco^2+^ are linked to IO_4_^−^ by six N-H···O hydrogen bonds, so none of the involved ions have extra charge and hydrogen atoms capable of forming strong attraction with water molecules, which may help explain why **DPPE-1** is able to exist as a stable material and precipitate easily from aqueous solutions, thus leading to simple synthesis and separation methods. The combination of stable chemical composition, abundant interionic interactions and strengthened three-dimensional framework is also likely to lead to a stable perovskite primary explosive.

### Stability studies

**Fig. 4:**
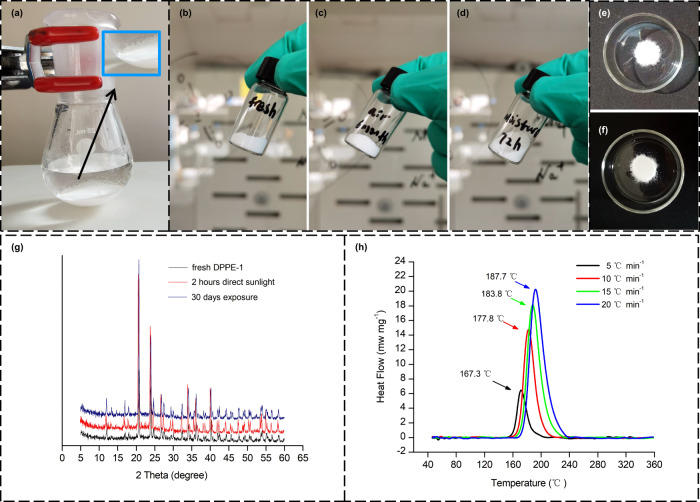
Stability of DPPE-1. **a** DPPE-1 precipitated from the solution. **b** Fresh DPPE-1. **c** Exposure to air for 6 months. **d** Exposure to moisture for several days. **e** Exposure to direct sunlight for 2 h. **f** Exposure to sunlight for 30 days. **g** Powder X-ray diffraction (PXRD) measurements in light stability tests. **h** DSC curves in thermal stability tests.

To verify whether **DPPE-1** is stable under ambient conditions, its stability towards air, moisture, sunlight, heat, and mechanical stimuli was investigated, along with its long-term storage stability. Considering that **DPPE-1** is synthesized, crystallized, and filtered from an aqueous solution, we conclude that it should be stable to air and moisture. Subsequent experiments confirmed that no matter that **DPPE-1** was exposed to air for 6 months or moisture for several days, no changes in color and morphology were observed (Fig. 4a–d). The chemical stability of **DPPE-1** was further verified by light stability test (Fig. 4e, f), which showed that **DPPE-1** remained in its original state after 2 h of direct sunlight and 30 days of sunlight exposure under ambient conditions, which was confirmed by powder X-ray diffraction analysis (Fig. 4g).

Differential scanning calorimetry (DSC) was used to assess the stability towards heat loading (details see Supplementary Information). Its thermal decomposition occurs with an onset temperature at 161.3 °C (Fig. 4h), comparable to dinitrodiazophenol (DDNP, 157.0 °C), 6-nitro-7-azido-pyrazol[3,4-d][1,2,3]triazine-2-oxide (ICM-103, 160.3 °C), and 6-azido-8-nitro-tetrazolo [1,5-b]pyridazine-7-amine (ANTPA, 163.0 °C)^19,20^, which satisfies most military and civilian needs. In addition, its thermal decomposition temperature is lower than that of reported perchlorate-based perovskite energetic materials (perchlorate-based, 179–371 °C)^25,47–50^, but it is comparable to four reported periodate-based analogues (152–169 °C)^42,43^. It should be noted that although **DPPE-1** meets the minimum thermal decomposition temperature requirements (*T*_dec_ ≥ 150 °C) of green primary explosives, it is more attractive to develop perovskite ignition materials with higher heat resistance (e.g. *T*_dec_ ≥ 180°C or even ≥ 200 °C)^4,10,52,53^. The long-term storage stability test was further performed by storing **DPPE-1** at atmospheric pressure and 75 °C for 48 h (Supplementary Table 9). The results showed that the mass loss of the **DPPE-1** was almost negligible (≤0.05%), suggesting excellent long-term storage stability.

In addition, we determined the mechanical sensitivity of **DPPE-1** (Supplementary Table 10). As evident from Table 1, its impact sensitivity (IS) and friction sensitivity (FS) are 3.5 J and 5.0 N, respectively. The impact sensitivity is comparable to those reported for most primary explosives, and the friction sensitivity is better than that of widely used military primary explosive LA^12^ and close to those reported periodate-based single perovskites used as biocidal agents (<5 N)^42^. Accordingly, it has acceptable mechanical sensitivity. The data in Table 1 also show that the oxygen balance (Ω_CO_ = −4.52%) of **DPPE-1** is higher than those of other initiating substances—possibly the highest oxygen balance for a primary explosive to date.

**Table 1:** Physical and energetic properties of several typical primary explosives and **DPPE-1**

Items	Formula	*M*^a^ (g mol^−1^)	Ω_CO_^b^ (%)	ρ^c^ (g cm^−3^)	IS^d^ (J)	FS^e^ (N)	*T*_dec_^f^ (^o^C)
**DPPE-1**	C_12_H_32_I_6_N_5_NaO_24_	1414.8	−4.52	2.88	3.5	5.0	161.3
LA^g^	PbN_6_	291.3	−5.49	4.80	2.5–4	0.1–1.0	315.0
SA^h^	AgN_3_	149.9	−21.35	4.81	>2.5	>0.1	>297.0
CA^i^	CuN_6_	147.6	−10.84	2.2-2.58	≤1.0	≤0.1	205.0
K_2_DNABT^j^	C_2_K_2_N_12_O_4_	291.3	0	2.11	1.0	≤1.0	200.0
K_2_DNAT^k^	CK_2_N_8_O_4_	266.3	−6.02	2.18	1.0	<5.0	240.0
K_2_BDAF^l^	C_6_HK_2_N_10_O_10_	450.3	+10.66	2.04	2.0	20.0	229.0
DDNP^m^	C_6_H_2_N_4_O_5_	210.1	−15.23	1.72	1.0	24.7	157.0
ICM-103^n^	C_4_HN_9_O_3_	223.1	−10.75	1.86	4.0	60.0	160.3
ANTPA^o^	C_4_H_2_N_10_O_2_	222.1	−21.61	1.82	5.0	120.0	163.0

*LA* lead azide, *SA* isilver azide. *CA* copper azide. See Fig. 1 for the chemical structures of other substances.

^a^formula weight. ^b^oxygen balance. ^c^crystal density. ^d^impact sensitivity. ^e^friction sensitivity. ^f^decomposition temperature, ^g^ref. ^10^, ^h^ref. ^1^, ^i^ref. ^1 & 7^, ^j^ref. ^10^, ^k^ref. ^12^, ^l^ref. ^14^, ^m^ref. ^20^, ^n^ref. ^20^, ^0^ref. ^21^.

### Ignition performance

**Fig. 5:**
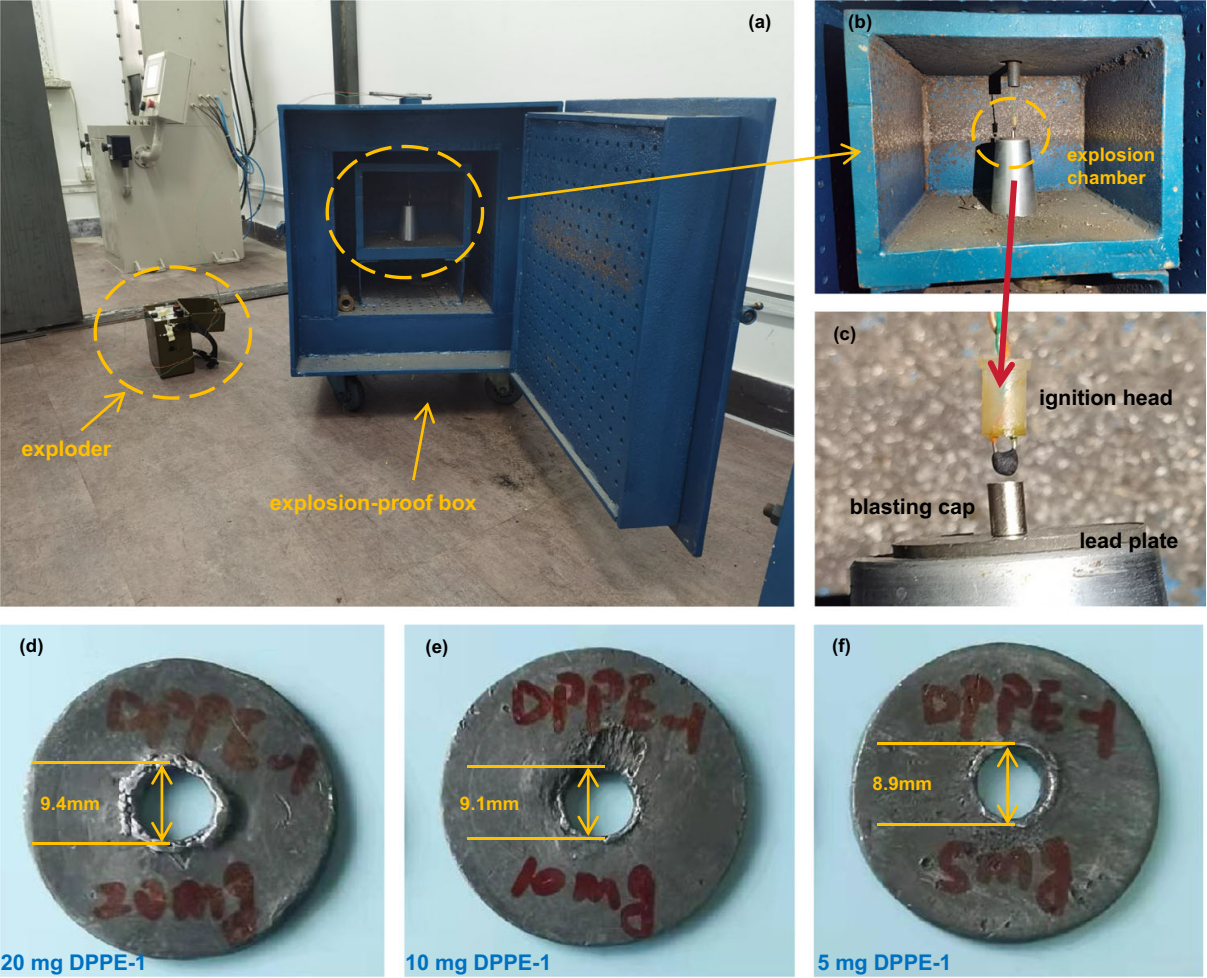
Evaluation of the ignition performance of the primary explosive DPPE-1. **a** the ignition performance test system. The exploder is a portable power supply, which is used to ignite the ignition head. **b** Explosion chamber. It is a metal box used to provide safety protection. **c** The combination of ignition head, detonator, and lead plate in the testing device. It is a key part for testing ignition performance. **d**–**f** Test results for the minimum primary charge (MPC).

The minimum primary charge (MPC) is the most important parameter for evaluating the ignition performance of a primary explosive. In this study, MPC was determined with the device shown in Fig. 5a–c (details see Supplementary Information 10. Ignition performance). The lead plate was successfully penetrated when the loading weight of DPPE-1 was 20 mg and 10 mg (Fig. 5d, e). We further reduced the loading weight to 5 mg, which is considered as the ultimate weight, because the surface of RDX cannot be completely covered if the amount of **DPPE-1** is less than 5 mg. Interestingly, 5 mg of **DPPE-1** is also able to initiate RDX reliably, making it an efficient initiating substance (Fig. 5f). It is clear that the ignition performance of **DPPE-1** is far superior to those of recently reported green primary explosives with claimed initiation efficiency (e.g. DDNP, ICM-103, ANTPA, K_2_DNABT, K_2_DNAT), and is comparable to those of most powerful primary explosives (e.g. PbN_6_, AgN_3_, CuN_6_) (Supplementary Table 11) and periodate-based perovskites^43^. However, **DPPE-1** can’t be a perfect replacement for PbN_6_ unless its thermal decomposition temperature is higher than 200 °C. As for K_2_DNABT, K_2_DNAT, and K_2_BDAF, they may be high-performance primary explosives, but the lengthy manufacturing process makes them almost impossible to replace PbN_6_. In industry, the excellent initiation performance of **DPPE-1** would enable excellent economic, social, and environmental benefits, contributing to a significant reduction in primary explosive production and lowering both environmental hazards and casualties.

How to explain the mechanism of such excellent ignition performance? Here, we calculate the heat of formation of **DPPE-1**, and then evaluate its energy level by using program EXPLO7.0 (Supplementary Table 12). The results show that its detonation velocity (D) and detonation pressure (P) are <5500 m/s and <18.5 GPa, respectively, which are lower than those of most primary explosives, indicating that the impressive initiation performance of **DPPE-1** is hard to be well explained from the energy level. So, we turned our attention to oxidation of IO_4_^-^. The ignition performance of the primary explosive is generally considered to depend mainly on the oxidation of the oxidizing components. For example, energetic substances based on perchlorate (ClO_4_^−^) are usually used to construct high-performance primary explosives^54,55^, with the reason that ClO_4_^−^ has stronger oxidation than nitrite (NO_3_^−^), chlorate (ClO_3_^−^), sulfate (SO_4_^2−^), styphnate (C_6_HN_3_O_8_^2−^), and so on, thus bringing excellent ignition performance. According to the Tables of Standard Electrode Potentials^56^, however, the standard electrode potential (*E*_*0*_) of IO_4_^−^ is 1.314 V, while that of ClO_4_^−^ is 1.389 V. Obviously, the oxidation of ClO_4_^−^ is stronger than that of IO_4_^-^, which demonstrates that the good initiation performance of **DPPE-1** may be related to some unknown influencing factors besides the strong oxidation of IO_4_^−^. We think that molecular stability is likely to play a crucial role, so we compare the Gibbs Free Energy (*ΔG*) of (H_2_dabco)_2_[Na(NH_4_)(IO_4_)_6_]_n_ and (H_2_dabco)_2_[Na(NH_4_) (ClO_4_)_6_]_n_ (Supplementary Table 13). The results show that the *ΔG* of (H_2_dabco)_2_[Na(NH_4_)(IO_4_)_6_] (*ΔG* = −144.95 kcal/mol) is higher than that of (H_2_dabco)_2_[Na(NH_4_)(ClO_4_)_6_] (*ΔG* = −201.98 kcal/mol), that is, the ClO_4_^−^ in (H_2_dabco)_2_[Na(NH_4_)(ClO_4_)_6_] has a good stabilizing effect, while the material based on IO_4_^−^ is relatively unstable, which will help us to understand the high mechanical sensitivity and strong initiation performance of **DPPE-1**. In any case, there is no doubt that halogens play a decisive role in the initiation performance of energetic perovskites according to our available studies. For example, compared with ClO_4_^−^-based materials, IO_4_^-^-based materials are more likely to exhibit high sensitivity, rapid exothermic processes, and strong initiation performance, while BrO_4_^−^-based materials are generally too unstable to be synthesize and used as energetic materials.

Furthermore, the explosive products of the perovskite primary explosive are discussed in detail through theory and experiment. It is well known that reducing the toxicity of explosive products is the main driving force to promote the development of green primary explosives. The rise of green primary explosive stems from the elimination of heavy metal toxicity from solid decomposition products (e.g. Pb and Hg) of early primary explosives^53^. Theoretically, highly sensitive substances without heavy metals Pb and Hg can be regarded as potential green primary explosives. This is an important reason why some substances are classified as green primary explosives even though they contain highly toxic components, including silver azide (SA), nickel hydrazine nitrate (NHN), copper(I) 5-nitrotetrazolate (DBX-1), bis-(5-nitrotetrazole)tetraamine cobalt(III) perchlorate (BNCP), 2-diazo-4,6-dinitrophenol (DDNP), cyanuric triazide (CTA), potassium 4,6-dinitro-7-hydroxybenzofuroxan (KDNP), and potassium 4,6-dinitrobenzofuroxan (KDNBF)^53^. Although the perchlorate-based primary explosives have reduced toxicity, their gaseous explosive product (e.g. HCl) is still uncomfortable. Component IO_4_^−^ is also biotoxic, however, its toxicity is at most comparable to the components such as hydrazine, azide, nitrotetrazolate, nitrophenol, benzofuroxan, and cyanuric triazide in the above-mentioned green primary explosives, and the main solid product (I_2_) of periodate-based primary explosives is a typical material with sterilization and disinfection functions. Therefore, **DPPE-1** should be considered as a non-toxic design ideas according to the current creteria. Energetic biocidal agents were put forward based on similar ideas^41,42^. Calculations based on the EXPLO7.0 program show that most of detonation products of **DPPE-1** are non-toxic and less toxic, with a mass percentage of I_2_ of 51.7% (Supplementary Table 14). To confirm the existence of I_2_ in the detonation product, we filled the **DPPE-1** in a pressure-resistant glass bottle and heated it in an oven to 200 °C As a result, we heard a huge explosion and the bottle was completely broken (Supplementary Fig. 12a, b); then we filled **DPPE-1** in a Teflon reactor and heated it in an oven to 200 °C. As a result, no explosion was heard and the detonation product stained the inner wall of the container purple, indicating the possible formation of I_2_ (Supplementary Fig. 12c, d). Further tests showed that the aqueous solution of the purple substance was yellow and immediately formed a blue solution when mixed with starch (Supplementary Fig. 12e–g), confirming the presence of a large amount of I_2_. So IO_4_^−^ is suitable for the construction of green primary explosives.

## Discussion

An interesting primary explosive (**DPPE-1**) has been reported, which consists of an organic–inorganic double perovskite structure reasonably different from the structure of traditional primary explosives. Its synthesis involves a simple green one-pot process and most of the decomposition products are non-toxic or less toxic, which makes this primary explosive both cost-effective and environmentally friendly. Single-crystal X-ray diffraction confirms its cubic structure to be similar to the well-known organic–inorganic hybrid perovskites formed through self-assembly of hierarchical structures. Detailed studies show that the primary explosive **DPPE-1** has an excellent comprehensive performance, such as high oxygen balance (−4.52%), high crystal density (2.88 g cm^−3^), high filling factor (80.7%), good environmental tolerance (to air, moisture, and light), reasonable thermal stability (*T*_dec_ = 161.3 °C), and acceptable mechanical sensitivity (IS = 3.5 J; FS = 5.0 N). Most impressive is its ultra-high initiation performance (MPC ≤ 5 mg). These factors led to the discovery of perovskite materials with ignition function, demonstrating that this organic–inorganic perovskite is an exceptional platform for developing advanced primary explosives. The search for perovskite-type green primary explosives with thermal decomposition temperatures higher than 200 °C or even 250 °C will be the focus of priority consideration in the future. Given the undoubted importance of perovskites and primary explosives in various critical engineering applications, the discovery of **DPPE-1** is likely to be a pioneer in materials science and engineering technology. It is expected that additional perovskite primary explosives or highly energetic perovskites will continue to be produced through independent structures or systematic combinations of organic and inorganic components.

## Methods

### Safety precautions

**DPPE-1** is a highly explosive, sensitive material, and it should be handled with extreme caution using proper safety equipment, such as protective gloves and coats, a face shield, and an explosion-proof baffle.

### Materials

Sodium metaperiodate (99.5%), ammonium chloride (99.8%), and triethylene diamine (98%) were purchased from Shanghai Aladdin Biochemical Technology Co., Ltd. Dabconium dihydrochloride was obtained by reacting triethylene diamine with dilute hydrochloric acid.

### Characterization

Infrared spectra (IR) were recorded on a Bruker Equinox 55 infrared spectrometer. Elemental analysis (C, H, and N) was performed on a varioMICRO cube fully automatic trace element analyzer. ^1^H and ^13^C NMR spectra were recorded on a Bruker Advance 600 nuclear magnetic resonance spectrometer. Powder X-ray diffraction (PXRD) measurements were performed on a Bruker D8 advance diffractometer. The single-crystal X-ray diffraction data were collected on a Rigaku AFC-10/Saturn 724 + CCD diffractometer. Thermal decomposition temperatures were determined using differential scanning calorimetry (DSC) on a CDR-4 from Shanghai Precision & Scientific Instrument Co. Ltd. The long-term storage stability and minimum primary charge (or initiation performance test) were measured according to the method given by GJB 5891-2006. The impact and friction sensitivity measurements were performed using a standard BAM Fall hammer and a BAM friction tester.

### Synthesis of DPPE-1

Dabconium dihydrochloride (0.37 g, 2 mmol) and ammonium chloride (0.0535 g, 1 mmol) were dissolved in 5 mL water by vigorous agitation (600 r min^−1^) at room temperature. Subsequent addition of 8 mL sodium metaperiodate (NaIO_4_, 1.28 g, 6 mmol) solution into the above mixture resulted in the immediate precipitation of a white solid and the reaction solution became clear and colorless within 2–3 s. The resulting precipitate was filtered, washed with an ice/water mixture (2 × 3 mL), and then sequentially dried under sunlight and vacuum to yield the target compound **DPPE-1** as a colorless solid. Yield: 1.02 g, 72.1%. DSC (5 °C min^−1^, °C): 161.3 (dec.); IR (KBr pellet, cm^−1^): ṽ 3122 (m), 3034 (w), 1475 (m), 1419 (s), 1328 (w), 1214 (m), 1056 (s), 830 (s). ^1^H NMR (600 MHz, DMSO-d_6_, 25 °C): *δ* = 7.06 ppm (1H, NH), 3.36 (2H, CH_2_); ^13^C NMR (600 MHz, DMSO-d_6_, 25 °C): *δ* = 43.90 ppm; EA calculated for C_12_H_32_I_6_N_5_NaO_24_ (1414.82 g mol^−1^): C 10.19, H 2.28, N 4.95; Found: C 10.14, H 2.19, N 4.89.

### Supplementary information

Supporting Information

Peer Review File

## Data Availability

Data that support the findings of this study are available from the corresponding authors on request. The supplementary crystallographic data generated in this study have been deposited in the Cambridge Crystallographic Data Centre (CCDC) database under accession code 2173977 via www.ccdc.cam.ac.uk/data_request/cif.
