# Supplementary material for: A promising perovskite primary explosive
Source: Nat Commun. 2023 Nov 27;14:7765. doi: 10.1038/s41467-023-43320-0 (PMC10681991; doi:10.1038/s41467-023-43320-0)
Supplement: Supplementary file 1 — Supporting Information [file 41467_2023_43320_MOESM1_ESM.pdf]

Supplementary Information

A Promising Perovskite Primary Explosive

Yongan Feng et al

Table of Contents

1. General information	3
2. Connection mode of periodate and cations	3
3. Synthesis	4
4. Fourier transform infrared spectroscopy (FTIR)	4
5. Nuclear magnetic resonance (NMR)	5
6. Crystallographic data	6-10
7. Filling coefficient	11
8. Thermal stability	11-12
9. Mechanical sensitivity	12- 13
10. Initiation performance	13-14
11. Detonation performance	14
12. Standard electrode potential and gibbs free energy	14
13. Explosive products	15-16
References	16-17

1.General information

All the materials and chemical reagents were analytical grade, purchased from Shanghai Aladdin Biochemical Technology Co., Ltd. and used without further purification. Infrared spectra (IR) are recorded on a Bruker Equinox 55 infrared spectrometer with KBr pellets from 400 to 4000 cm^{-1} with a resolution of 4 cm^{-1}. Elemental analyses (C, H, and N) were performed on a varioMICRO cube fully automatic trace element analyzer. ^{1}H and ^{13}C NMR spectra were recorded on a Bruker Advance 600 nuclear magnetic resonance spectrometer by using DMSO-d_6 as solvent. Powder X-ray diffraction (PXRD) measurements were performed on a Bruker D8 advance diffractometer at 60 kV, 300 mA and Cu Kα radiation (λ = 1.5406 Å) with a scan speed of 5 °·min^{-1} and a step size of 0.02° in 2θ. The single-crystal X-ray diffraction data collections were carried out on a Rigaku AFC-10/Saturn 724+CCD diffractometer with graphite-monochromated Mo Kα radiation (λ = 0.71073 Å) using a multi-scan technique. The crystal structure was determined by direct methods using SHELXS-97 and refined by full-matrix least-squares procedures on F_2 with SHELXL-97.[1-2] All non-hydrogen atoms were obtained from the difference Fourier map and subjected to anisotropic refinement by full-matrix least squares on F_2. Hydrogen atoms were obtained geometrically and treated as riding on the parent atoms or were constrained in the locations during refinements. The thermal decomposition temperatures were determined by using differential scanning calorimetry (DSC) on a CDR-4 of Shanghai Precision & Scientific Instrument Co., Ltd. at heating rate of 5 ℃·min^{-1} from 40 ℃ to 350 ℃ under a dry oxygen-free nitrogen atmosphere with a flowing rate of 20 mL·min^{-1}. The long-term storage stability and minimum primary charge test (or ignition capacity test) were measured according to the method given by GJB 5891-2006 (the related standard documents can be obtained through websites http://www.rongrong.cn).[3] Impact and friction sensitivity measurements were made using a standard BAM Fall hammer and a BAM friction tester.

2.Connection mode of periodate and cations

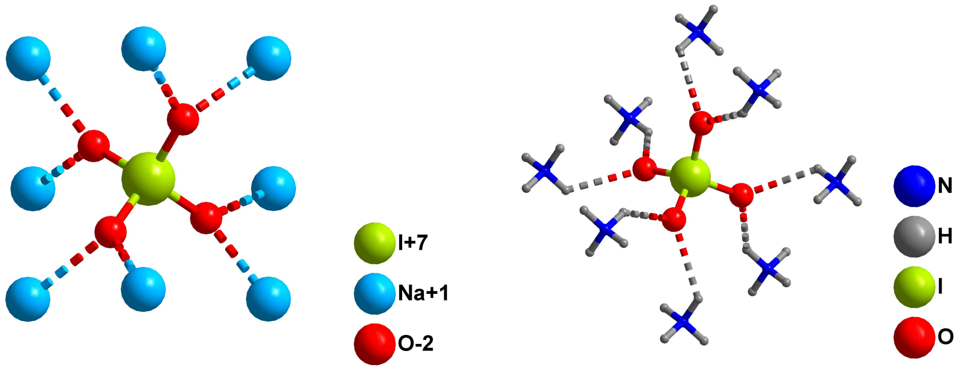

Supplementary Fig. 1 Interactions between IO_4^-, Na^+, and NH_4^+.

3.Synthesis

The typical synthesis process was as follows. Dabconium dihydrochloride (DABCO, 0.37 g, 2 mmol) and ammonium chloride (NH_4Cl, 0.0535 g, 1 mmol) were added to 5 mL water and dissolved with vigorous agitation (600 $r{\cdot}min^{-1}$) at room temperature. Sodium metaperiodate ($NaIO_4$, 1.28 g, 6 mmol) in 8 mL water was poured into the mixture, and a large amount of white solid rapidly precipitated from the clear colorless solution within 2~3 seconds. The resulting precipitate was filtered and washed with ice/water mixture (2 × 3 mL). The solid product was exposed to the direct sunlight for 2 hours, and dried at 60 ℃ for 2 hours to give the colorless **DPPE-1**. Yield: 1.02 g, 72.1%. DSC (5 $℃{\cdot}min^{-1}$,): $T_{d\ (onset)}$ = 161.3 ℃ (dec.); IR (KBr pellet, cm^{-1}): 3122 (m), 3034 (w), 1475 (m), 1419 (s), 1328 (w), 1214 (m), 1056 (s), 830 (s). ^{1}H NMR (600 MHz, DMSO-d_6, 25 °C): δ = 7.06 ppm (1H, NH), 3.36 (2H, CH_2); ^{13}C NMR (600 MHz, DMSO-d_6, 25 ℃): δ = 43.90 ppm; EA calculated for $C_{12}H_{32}I_6N_5NaO_{24}$ (1414.82 $g{\cdot}mol^{-1}$): C 10.19, H 2.28, N 4.95; Found: C 10.14, H 2.19, N 4.89.

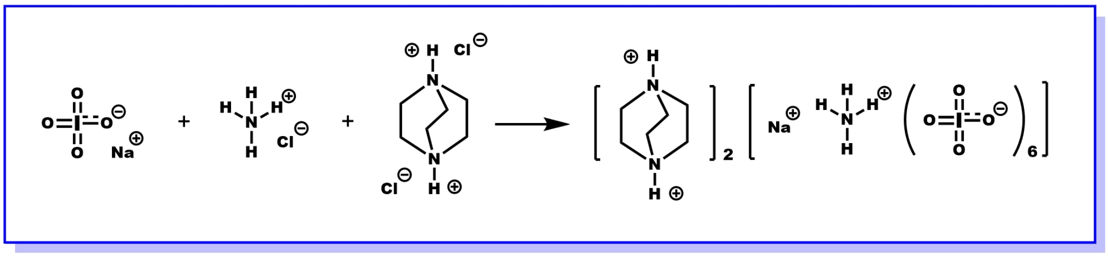

Supplementary Fig. 2 The synthesis of **DPPE-1**.

4.Fourier transform infrared spectroscopy (FTIR)

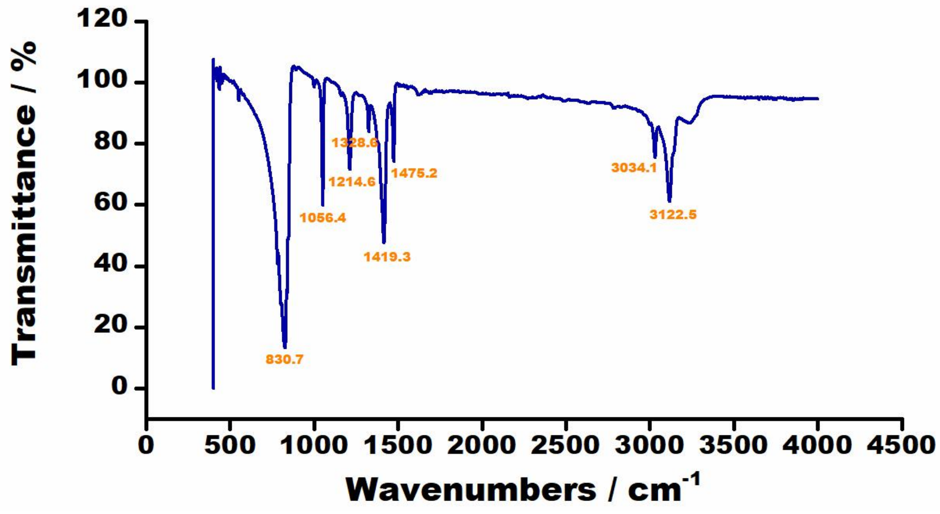

Supplementary Fig. 3 The infrared spectrum of **DPPE-1**.

5. Nuclear magnetic resonance (NMR)

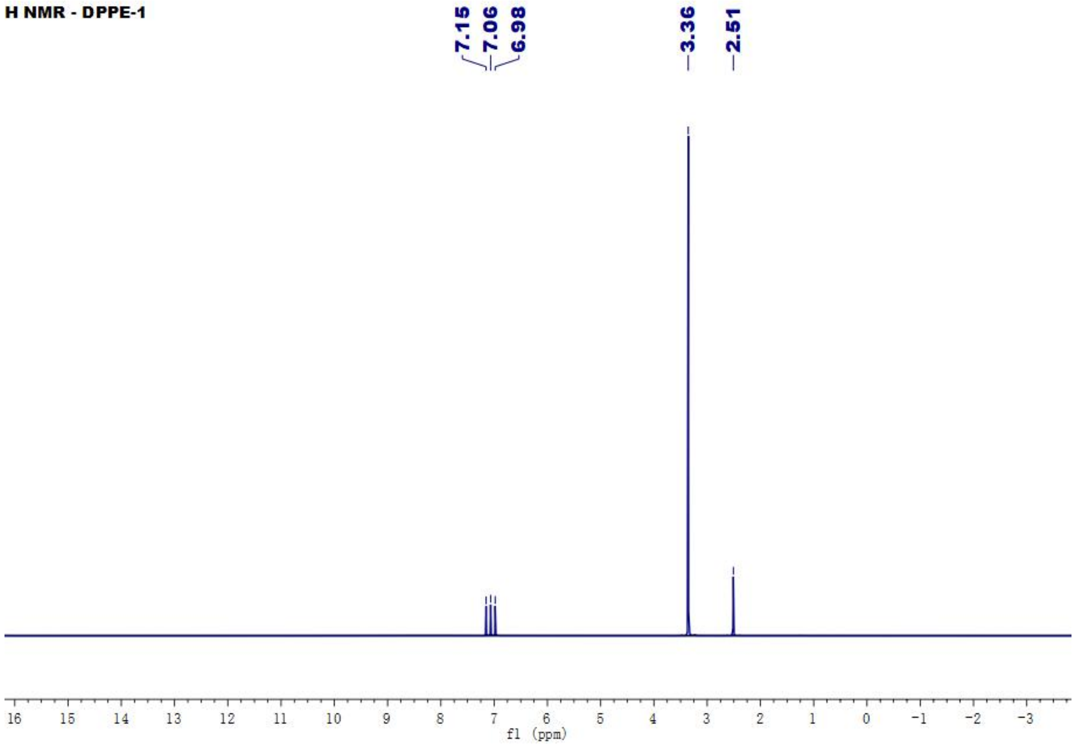

Supplementary Fig. 4 The ^{1}H NMR spectrum of **DPPE-1**.

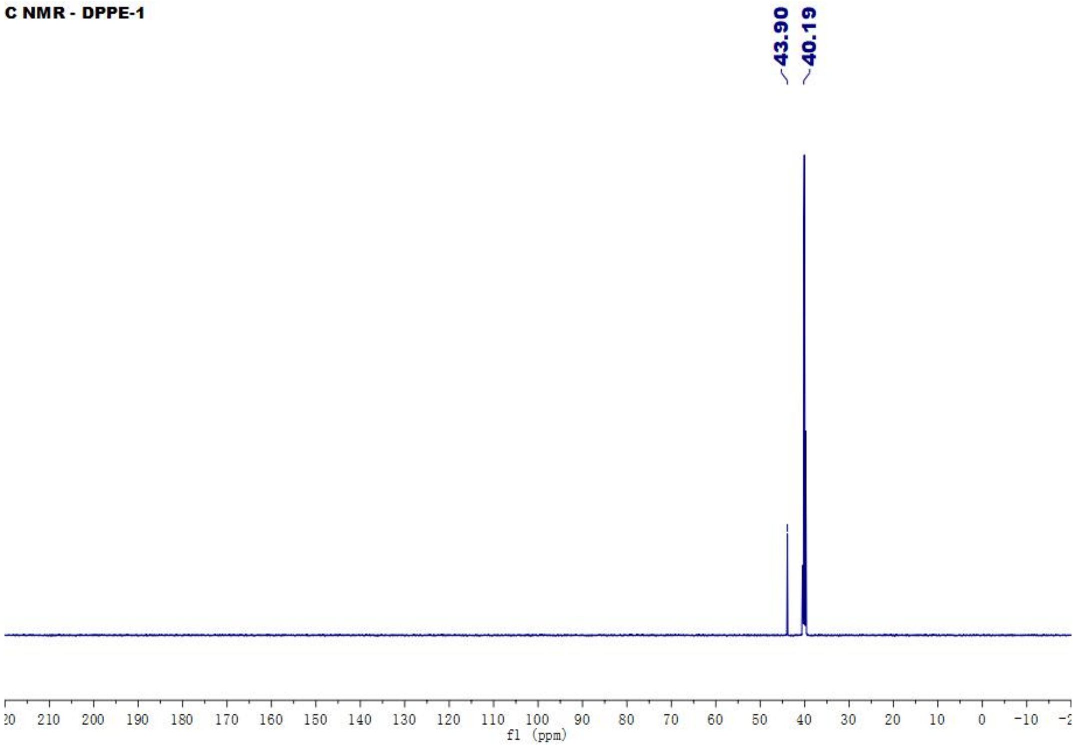

Supplementary Fig. 5 The ^{13}C NMR spectrum of **DPPE-1**.

6.Crystallographic data

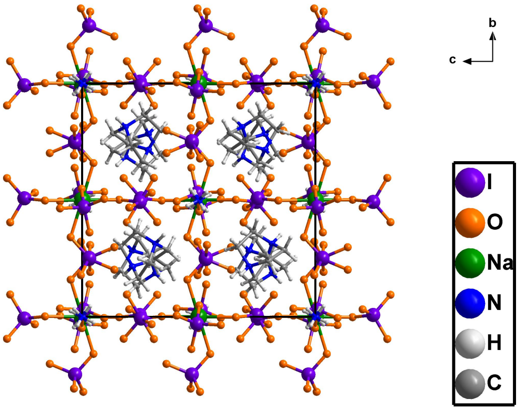

Supplementary Fig. 6 The crystal stacking structure of **DPPE-1** seen from a axis.

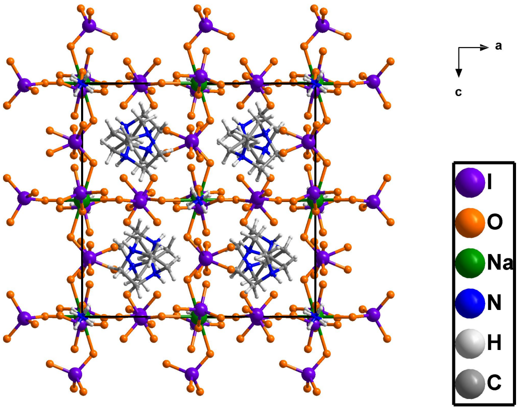

Supplementary Fig. 7 The crystal stacking structure of **DPPE-1** seen from b axis.

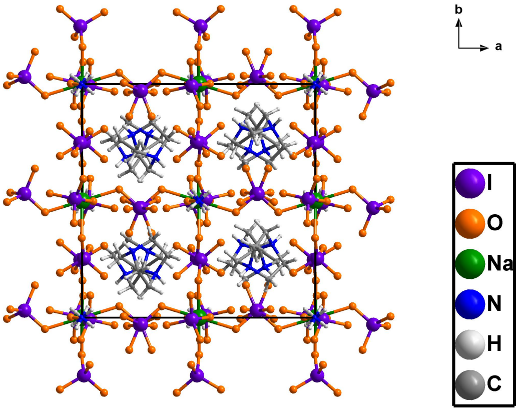

Supplementary Fig. 8 The crystal stacking structure of **DPPE-1** seen from c axis.

Supplementary Table 1 Crystal data and structure refinement for **DPPE-1**.

Compound Name	DPPE-1
Empirical formula	$C_{12}H_{32}I_6N_5NaO_{24}$
Formula weight	1414.82
Temperature / K	120.2(3)
Crystal system	Cubic
Space group	Pa-3
a / Å, b / Å, c / Å	14.8167(2), 14.8167(2), 14.8167(2)
α/°, β/°, γ/°	90.00, 90.00, 90.00
Volume / Å^3	3252.78(8)
Z	4
ρ_{calc} / mg mm^{-3}	2.889
μ / mm^{-1}	5.845
F(000)	2640
Crystal size / mm^3	0.21 × 0.21 × 0.18
2Θ range for data collection	3.07 to 24.95°
Index ranges	-13 ≤ h ≤ 17, -17 ≤ k ≤ 17, -17 ≤ l ≤ 17
Reflections collected	11373
Independent reflections	3152[R(int) = 0.0435 (inf-0.9Å)]
Data/restraints/parameters	961/0/75
Goodness-of-fit on F^2	1.069
Final R indexes [I>2σ (I) i.e. F_o>4σ (F_o)]	R_1 = 0.0231, wR_2 = 0.0472
Final R indexes [all data]	R_1 = 0.0262, wR_2 = 0.0483
Largest diff. peak/hole / e Å^{-3}	1.122/-1.010
Flack Parameters	N
Completeness	0.9964

Supplementary Table 2 Bond lengths for **DPPE-1**.

Atom	Atom	Length/Å	Atom	Atom	Length/Å
I1	O4	1.773(4)	O9	Na1	2.463(4)
I1	O2	1.770(4)	Na1	O11	2.462(5)
I1	O3	1.770(4)	Na1	O51	2.464(4)
I1	O1	1.757(4)	Na1	O91	2.463(4)
O1	Na1	2.462(5)	N1	C4	1.495(7)
I2	O7	1.769(3)	N1	C5	1.497(6)
I2	O8	1.765(4)	N1	C1	1.483(7)
I2	O6	1.777(4)	C2	C1	1.533(8)

[1]1-X,1-Y,1-Z

Supplementary Table 3 Bond lengths for **DPPE-1** (continue).

Atom	Atom	Length (Å)	Atom	Atom	Length (Å)
I2	O5	1.760(4)	C2	N2	1.503(7)
O5	Na1	2.464(4)	C3	C4	1.532(8)
I3	O12	1.773(4)	C3	N2	1.503(7)
I3	O11	1.757(4)	C6	C5	1.535(8)
I3	O10	1.774(4)	C6	N2	1.503(7)
I3	O9	1.759(4)			

[1]1-X,1-Y,1-Z

Supplementary Table 4 Bond angles for **DPPE-1**.

Atom	Atom	Atom	Angle/°	Atom	Atom	Atom	Angle/°
O2	I1	O4	110.81(17)	O1[1]	Na1	O5	106.71(13)
O2	I1	O3	108.77(19)	O1	Na1	O5	73.29(13)
O3	I1	O4	109.16(18)	O1	Na1	O9[1]	73.46(14)
O1	I1	O4	109.3(2)	O1[1]	Na1	O9[1]	106.54(14)
O1	I1	O2	109.01(17)	O1[1]	Na1	O9	73.46(14)
O1	I1	O3	109.7(2)	O1	Na1	O9	106.54(14)
I1	O1	Na1	121.3(2)	O5[1]	Na1	O5	180.0
O7	I2	O6	110.77(18)	O9[1]	Na1	O5	106.62(13)
O8	I2	O7	108.78(18)	O9	Na1	O5	73.38(13)
O8	I2	O6	109.35(19)	O9	Na1	O5[1]	106.62(13)
O5	I2	O7	108.86(18)	O9[1]	Na1	O5[1]	73.38(13)
O5	I2	O8	109.7(2)	O9[1]	Na1	O9	180.0
O5	I2	O6	109.4(2)	C4	N1	C5	109.7(4)
I2	O5	Na1	121.0(2)	C1	N1	C4	110.1(4)
O12	I3	O10	110.87(18)	C1	N1	C5	109.9(4)
O11	I3	O12	108.89(19)	N2	C2	C1	108.2(4)
O11	I3	O10	108.88(18)	N2	C3	C4	108.3(4)
O11	I3	O9	109.8(2)	N2	C6	C5	108.0(4)
O9	I3	O12	108.94(17)	N1	C4	C3	108.1(5)
O9	I3	O10	109.4(2)	N1	C5	C6	108.3(4)
I3	O9	Na1	121.1(2)	N1	C1	C2	108.5(4)
O1[1]	Na1	O1	180.000(1)	C3	N2	C2	109.2(4)
O1[1]	Na1	O5[1]	73.29(13)	C6	N2	C2	109.8(4)
O1	Na1	O5[1]	106.71(13)	C6	N2	C3	109.5(4)

[1]1-X,1-Y,1-Z

Supplementary Table 5 Torsion angles for **DPPE-1**.

Bond	Torsion angle [°]	Bond	Torsion angle [°]	Bond
I1	O1	Na1	O1[1]	161(100)
I1	O1	Na1	O5	90.0(2)
I1	O1	Na1	O5[1]	-90.0(2)
I1	O1	Na1	O9	23.6(2)
I1	O1	Na1	O9[1]	-156.4(2)
O4	I1	O1	Na1	7.4(3)
O2	I1	O1	Na1	128.65(19)
O3	I1	O1	Na1	-112.3(2)
I2	O5	Na1	O1[1]	-23.4(3)
I2	O5	Na1	O1	156.6(3)
I2	O5	Na1	O5[1]	-4(100)
I2	O5	Na1	O9[1]	90.1(2)
I2	O5	Na1	O9	-89.9(2)
O7	I2	O5	Na1	-128.7(2)
O8	I2	O5	Na1	112.4(2)
O6	I2	O5	Na1	-7.5(3)
I3	O9	Na1	O1	-90.1(2)
I3	O9	Na1	O1[1]	89.9(2)
I3	O9	Na1	O5	-156.4(3)
I3	O9	Na1	O5[1]	23.6(3)
I3	O9	Na1	O9[1]	-129(100)
O12	I3	O9	Na1	128.7(2)
O11	I3	O9	Na1	-112.1(2)
O10	I3	O9	Na1	7.3(3)
N2	C2	C1	N1	-15.6(6)
N2	C3	C4	N1	-16.3(6)

[1]1-X,1-Y,1-Z

Supplementary Table 6 Hydrogen Bonds for **DPPE-1**.

Bond	X-H [Å]	H···Y [Å]	X···Y [Å]	Angle [°]
N1-H1··· O12	0.91	2.34	3.007(6)	130
N1-H1···O2	0.91	2.35	3.009(5)	129
N1- H1···O7	0.91	2.35	3.011(6)	129
N2-H2···O6	0.91	2.31	2.973(6)	129
N2- H2···O4	0.91	2.32	2.978(6)	129
N2- H2··· O10	0.91	2.32	2.975(6)	129
N3-H3C···O11	0.91	2.53	3.223(4)	133
N3-H3C···O2	0.91	2.24	2.990(4)	139
N3-H3D···O7	0.91	2.31	2.992(3)	131
N3-H3D···O3	0.91	2.46	3.219(4)	141
N3- H3E···O12	0.91	2.35	2.990(3)	127
N3- H3E···O8	0.91	2.42	3.218(4)	146
N3-H3F···O12	0.91	2.27	2.990(3)	136
N3- H3F···O2	0.91	2.39	2.990(4)	123
N3-H3F···O7	0.91	2.35	2.992(3)	128
C1-H1A···O4	0.97	2.47	3.442(7)	177
C1-H1B···O1	0.97	2.41	3.346(7)	162
C2-H2B···O7	0.97	2.57	3.351(7)	138
C3-H3A···O3	0.97	2.60	3.499(6)	155
C3-H3B···O2	0.97	2.56	3.354(6)	139
C4-H4A···O10	0.97	2.47	3.436(7)	178
C4-H4B···O9	0.97	2.41	3.344(7)	162
C5-H5A···O6	0.97	2.47	3.438(7)	177
C5-H5B···O5	0.97	2.41	3.343(7)	162
C6-H6A···O11	0.97	2.60	3.501(7)	155
C6-H6B···O12	0.97	2.56	3.347(6)	139

7.Filling coefficient

```
:: *   scratch :  400MB   *  HELP for Available Instruction Information
:: ************************  PLUTON to enter the PLUTON sub-program
>>calc void

:: TITL exp_8022         P c a b      R = 0.03
:: LAMBDA    0.71073
:: CELL     14.8167   14.8167   14.8167    90.000    90.000    90.000    3252.8
:: SPGR P c a b
:: Resd  1, SOF 1.000, Z  4, I6 Na O24
:: Resd  2, SOF 1.000, Z  8, C6 H14 N2
:: Resd  3, SOF 1.000, Z  4, H4 N
:: Moiety_Formula = I6 Na O24, 2(C6 H14 N2), H4 N
:: Sum_Formula    = C12 H32 I6 N5 Na O24
:: Formula_Weight =      1414.82 [Note: Based on SHELXL2014 Atomic Weights]
:: Formula_Z      =            4
:: SpaceGroup_Z   =            8
:: Formula_Z'     =        0.500
:: mu(MoKa)       =        58.45 cm-1 =    5.845 mm-1
:: Predicted Vol  =       3858.2[    3793.0] Ang**3, 298[120]K

:: VOID/SOLV Gridstep (Angstrom) (re)set to 0.20, Percent Memory =   0.4

van der Waals (or ion) Radii used in the Analysis
================================================================================
    C    H    I    N   Na    O
--------------------------------------------------------------------------------
 1.70 1.20 1.98 1.55 2.27 1.52

:: Nr of VOID Grid-points =       0, Percent Filled Space 80.7 (= Packing Index)

:: Unit cell Contains NO Residual Solvent Accessible Void.
```

Supplementary Fig. 9 Calculated filling coefficient (or packing coefficient) of **DPPE-1**.

8.Thermal stability

(1) Thermal decomposition

The thermal decomposition behavior of **DPPE-1** is studied using differential scanning calorimetry (DSC) at a linear heating rate of 5 $^{o}C\cdot min^{-1}$, 10 $^{o}C\cdot min^{-1}$, 15 $^{o}C\cdot min^{-1}$ and 20 $^{o}C\cdot min^{-1}$, respectively. The curves are shown in Supplementary Fig. 10. In addition, the kinetics parameters, including apparent activation energy (E_a) and the pre-exponential factor (A), was calculated by using Kissinger's method and Ozawa-Doyle's method according to the peak temperatures of first decomposition processes shown in the DSC curves [4-5].

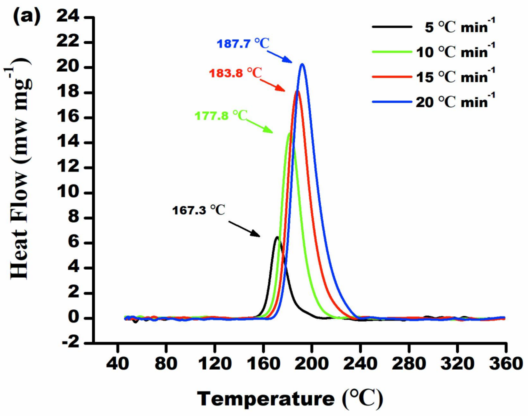

Supplementary Fig. 10 DSC curves of **DPPE-1** at different heating rates.

Supplementary Table 7 The measured peak temperatures of **DPPE-1**.

Heating rate [℃ min^{-1}]	Peaks temperatures [℃]
5	167.3
10	177.8
15	183.8
20	187.7

Supplementary Table 8 The calculated kinetics parameters of **DPPE-1**.

Items	Kissinger's method	Ozawa-Doyle's method
$E_{k/o}$ [kJ mol^{-1}]	106.4	108.3
Log A [s^{-1}]	10.36	
Linear correlation coefficient (R_k)	-0.9994	-0.9995
r	2.341e-002	1.03e-002
E_a [kJ mol^{-1}]	107.4	

(2) Long-term thermal stability

Two grams of **DPPE-1** samples were maintained at 75 ℃ for 48 h. The mass loss of the samples was measured, and the long-term thermal stability of **DPPE-1** was evaluated by the mass fraction of the sample loss.

Supplementary Table 9 The results of the long-term thermal stability test of **DPPE-1**.

Item	No.1	No.2	Average
mass loss of the sample [mg]	0.8	0.7	0.75
mass fraction of the sample loss [%]	0.040	0.035	0.0375

9.Mechanical sensitivity

The impact and friction sensitivities were assigned according to the UN recommendations on the transport of dangerous goods [6]. The sensitivities against impact (IS) and friction (FS) were determined according to BAM standards using a BAM drop hammer and a BAM friction apparatus.[7]

Supplementary Table 10 Measured sensitivities of LA, DDNP, ICM-103 and **DPPE-1**.

Items	IS (J)	FS (N)
DPPE-1	3.5	5.0
LA	4.0	1.0
DDNP	1.0	20
ICM-103	4.0	60

10.Ignition performance

The ignition performance determines the ability of a primary explosive to initiate secondary explosives, and was assessed by the minimum primary charge (MPC), which was conducted by the detonation against a lead plate (thickness: 5 mm) using 1,3,5-trinitro-1,3,5-triazacyclohexane (RDX) as a secondary explosive and defined by a critical weight, below which the lead plate cannot be blasted out of the hole. The ignition performance test system is shown below (Supplementary Fig. 11*a*-*c*). The chemicals involved in the experiment include lead styphnate (LTNR), 1,3,5-trinitro-1,3,5-triazacyclohexane (RDX) and **DPPE-1**.

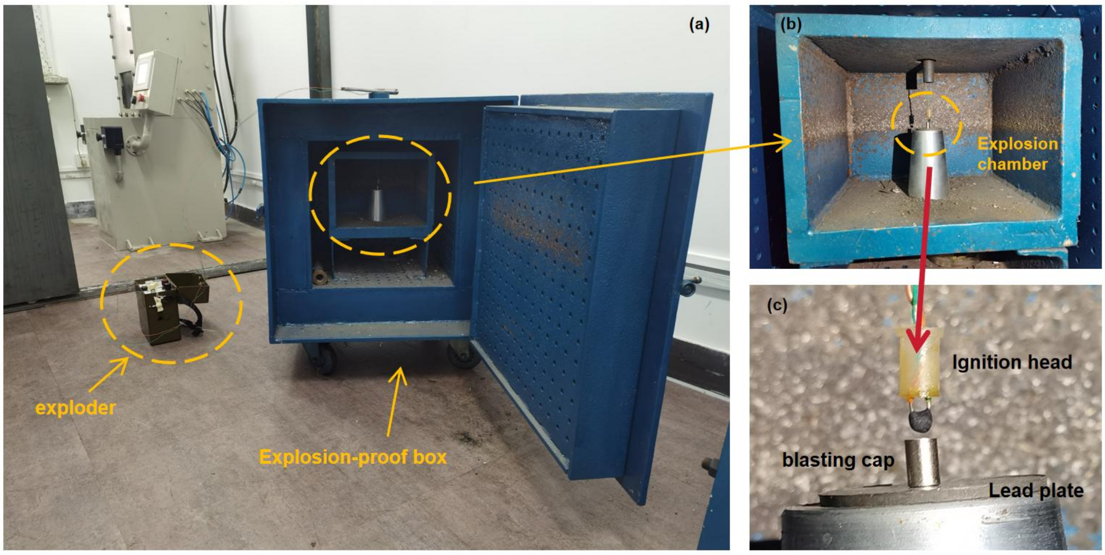

Supplementary Fig. 11 (a) Ignition performance test system; (b) Explosion chamber; (c) The combination of ignition head, blasting cap, and lead plate in the testing device.

In this test, the minimum primary charge test of **DPPE-1** toward 1,3,5-trinitro-1,3,5-triazacyclohexane (RDX) is carried out by using a standard blasting cap (Supplementary Fig. 11*c*). First, LTNR (30mg), **DPPE-1** (5~20mg), and RDX (20mg) are pressed successively into a standard blasting cap (I) with a pressure of 49.0 MPa. Then, the RDX (30mg) was pressed into a standard blasting cap (II) with a pressure of 117.6 MPa and a

layer of uncompacted RDX was added to the compacted RDX surface. Finally, standard blasting cap (I) was pressed into standard blasting cap (II) at a pressure of 49.0 MPa, then their combination was used to test the initiation performance.

Supplementary Table 11 The minimum primary charges (MPCs) of some reported primary explosives.

Compound	Minimum Primary Charges (mg)	Description
DDNP	70	Pure DDNP, filled in a No. 8 blasting cap and pressed with a static pressure of 32 MPa
ICM-103 [8]	60	Pure ICM-103, filled in a No. 8 blasting cap and pressed with a static pressure of 32 MPa
ANTPA [9]	40	Pure ANTPA, pressed with a static pressure of 40 MPa
K_2DNABT [10]	≤40	Pure K_2DNABT, steel and aluminum block setup
K_2DNAT [11]	≤50	Pure K_2DNAT, copper plate
K_2BDAF	not available	
PbN_6 (LA)[12]	40	Pure LA, measured in 6 mm copper tube
AgN_3 (SA)[13]	10~20	AgN_3 samples after high temperature stroage (220℃，100h)
CuN_6 (CA)	≤5	MPC is not available, and is inferred from some Ca-based substances [14-16]

11.Detonation performance

Supplementary Table 12 Detonation performance of DPPE-1.

Items	DPPE-1	
Temperature (T) / K	173	298
Density (d) / g cm^{-3}	2.88	2.74
Heat of formation (ΔH) / kJ mol^{-1}	-2928.80	-2928.80
Detonation velocity (D) / m s^{-1}	5403	5156
Detonation pressure (P) / GPa	18.2	16.4

The above calculation is completed through the EXPLO7.0 program

12.Standard electrode potential and gibbs free energy

Supplementary Table 13 The standard electrode potential and gibbs free energy.

Compounds	$(H_2dabco)_2[Na(NH_4)(IO_4)_6]$	$(H_2dabco)_2[Na(NH_4)(ClO_4)_6]$
Electrode reaction	$2IO_4^- + 16H^+ + 14e \rightleftharpoons I_2 + 8H_2O$	$ClO_4^- + 8H^+ + 8e \rightleftharpoons Cl^- + 4H_2O$
Standard electrode potential (E_0) / V	1.314	1.389
Gibbs free energy (ΔG) / kcal mol^{-1}	-144.95	-201.98

E_0, Refer to literature “G. Milazzo et al 1978 J. Electrochem. Soc. 125 261C”;
ΔG, obtained through theoretical calculations.

13.Explosive products

Calculations based on the EXPLO 7 program show that most of detonation products of DPPE-1 are non-toxic and less toxic, with a mass percentage of I2 of 51.7% (Supplementary Table 14). To confirm the I_2 in the detonation product, (a) we filled the DPPE-1 in a pressure-resistant glass bottle and heated it in an oven to 200 ℃ As a result, we heard a huge explosion and the bottle was completely broken (Supplementary Fig. 12a-b); (b) we filled DPPE-1 in a Teflon reactor and heated it in an oven to 200 ℃. As a result, no explosion was heard and the detonation product stained the inner wall of the container purple, indicating the possible formation of I_2 (Supplementary Fig. 12c-d). Further tests showed that the aqueous solution of the purple substance was yellow and immediately formed a blue solution after mixing with starch (Supplementary Fig. 12e-g), confirming the presence of I_2. The content of I_2 based on the color comparison method is > 50%, which is consistent with the theoretical calculation.

Supplementary Table 14 The detonation products of DPPE-1 calculated based on the EXPLO 7 program.

Detonation product	Mass percentage / %
H_2O	16.8177
C	5.3325
**I_2**	**51.7077**
CO_2	8.7824
N_2	4.6804
CH_2O_2	5.0482
CO	1.0721
Na_2CO_3	3.7457
NH_3	0.3267
CH_4	0.2729
HI	2.1273
H_2	0.0206
C_2H6	0.0603
C_2H_4	0.0032
HCN	0.0017
CH_3OH	0.0013

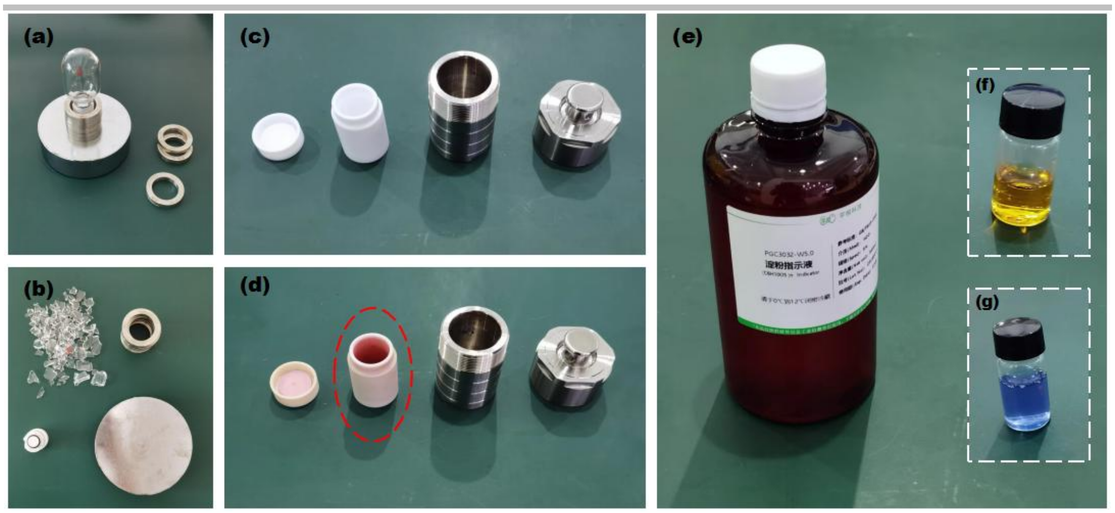

Supplementary Fig. 12 Experiment of detecting iodin (I2) in explosive products.

Supplementary References

1. Sheldrick, G. M. SHELXS-97, Program for X-ray Crystal Structure Determination, University of Göttingen, Germany, 1997.
2. Sheldrick, G. M. SHELXL-97, Program for X-ray Crystal Structure Refinement, University of Göttingen, Germany, 1997.
3. National Military Standard of the People's Republic of China, Test method of loading material for initiating explosive device, *GJB 5891-2006* (2005).
4. Kissinger, H. E. *Anal. Chem.*, **29**, 1702-1706 (1957).
5. Ozawa, T. *Bull. Chem. Soc. Jpn.*, **38**, 1881-1886 (1965).
6. Transport of Dangerous Goods, Manual of Tests and Criteria, 4th ed, United Nations, New York -Geneva, 1999.
7. Bundesanstalt für Materialforschung und-prüfung, http:// www.bam.de (accessed December 25, 2012).
8. Deng, M.; Feng, Y.; Zhang, W.; Qi, X. & Zhang, Q. *Nat. Commun.* **10**, 1339 (2019).
9. Huang, W.; Tang, Y.; Imler, G. H.; Parrish, D. A. & Shreeve J. M. *J. Am. Chem. Soc.* **142**, 3652-3657 (2020).
10. Fischer, D.; Klapötke, T. M. & Stierstorfer, J. *Angew. Chem. Int. Ed.* **53**, 8172-8175 (2014).
11. Fischer, D.; Klapötke, T. M. & Stierstorfer, J. *Angew. Chem. Int. Ed.* **54**, 10299-10302 (2015).
12. Matyáš, R. & Pachman, J. *Primary Explosives* Ch. 4, 79-79 (Springer, Heidelberg, 2013).
13. Liu, L.; Sheng, D.; Zhu, Y. & Dong, L. Research on Thermostable Performance of Silver Azide. *Initiators & Pyrotechnics*. **3**, 1003-1480 (2018).

14. Wurzenberger, M. H. H.; Lommel, M.; Gruhne, M. S.; Szimhardt, N. & Stierstorfer, J. Refinement of copper(Ⅱ) azide with 1-alkyl-5h-tetrazoles: adaptable energetic complexes. *Angew. Chem. Int. Edit.* **59**, 12367-12370 (2020).
15. Feng, Y.; Chen, S.; Li, Z. & Zhang, T. Performance of copper(II)-azide with hydrogen bonding as initiating explosive. *Chem. Eng. J.* **429**, 132186 (2022).
